# Early diagnosis of coronary heart disease based on retinal microvascular parameters of OCTA

**DOI:** 10.3389/fcell.2025.1654159

**Published:** 2025-08-12

**Authors:** Shijia Zhou, Qifeng Yan, Jiaqi Guo, Wenming He, Yitian Zhao

**Affiliations:** ^1^ Laboratory of Advanced Theranostic Materials and Technology, Ningbo Institute of Materials Technology and Engineering, Chinese Academy of Sciences, Ningbo, China; ^2^ University of Chinese Academy of Sciences, Beijing, China; ^3^ Cardiovascular Department, The First Affiliated Hospital of Ningbo University, Ningbo, China

**Keywords:** coronary artery disease, retinal vasculature, optical coherence tomography angiography, retinal microvascular parameters, biomarkers

## Abstract

Coronary artery disease (CAD) is a leading cause of cardiovascular mortality worldwide, and its early diagnosis is essential for prevention and treatment. Emerging evidence from ocular imaging suggests that structural and functional alterations in the retinal vasculature may mirror systemic vascular changes, offering a promising avenue for the early identification of cardiovascular conditions such as CAD. Among these techniques, OCTA stands out as a non-invasive, high-resolution modality capable of capturing detailed microvascular architecture and quantifying retinal blood flow dynamics. In this study, we analyzed OCTA images from 747 participants including 332 patients with CAD and 415 controls to extract retinal microvascular parameters and evaluate their associations with disease status. The results revealed that patients with CAD exhibited significantly lower retinal vessel density, vessel length density, tortuosity, and vascular bifurcation complexity on OCTA compared to controls, particularly in the left eye. This suggests possible lateral asymmetry in microvascular responses associated with CAD. Overall, we highlight the potential of OCTA-derived retinal biomarkers in supporting the early diagnosis of CAD, providing a standard tool for future clinical services and research. Biomarkers in retinal OCTA images can provide useful information for clinical decision making and diagnosis of CAD.

## 1 Introduction

**FIGURE 1:**
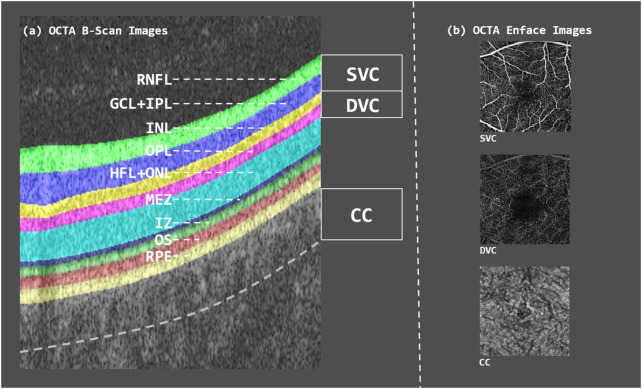
B-scan image of OCTA volume as well as en-face contrast of each layer. **(a)** OCTA B-scan image showing the segmented retinal layers, each highlighted in different colors. **(b)** OCTA en-face images of the three vascular layers—SVC, DVC, and CC.

CAD is characterized by an inadequate supply of blood and oxygen to the myocardium, primarily caused by the narrowing or blockage of the coronary arteries due to plaque buildup. This imbalance between oxygen supply and myocardial demand significantly contributes to the development of CAD, which has become a leading cause of mortality worldwide (Zaman et al., 2025; Camaj et al., 2025; Attiq et al., 2024). Early screening and prevention among high-risk populations are crucial strategies for reducing the incidence of CAD (Tsao et al., 2023; Libby and Theroux, 2005; Shao et al., 2020; Malakar et al., 2019). The retinal and coronary vasculature share similar anatomical and physiological properties. Notably, retinal vessel offer a unique, non-invasive window into vascular health, as they are the only microvasculature in the body that can be directly observed. This makes them valuable for studying CAD (Williams et al., 2004; Wong and Mitchell, 2004; Wong et al., 2001). Retinal imaging is currently applied in two main areas within cardiovascular research. First, it is used to investigate the role and underlying mechanisms of the microvascular system in the progression of cardiovascular diseases. Second, it serves as a potential clinical tool for risk stratification, helping clinicians identify individuals at elevated risk for cardiovascular and cerebrovascular events based on microvascular changes (Touyz, 2007). OCTA is an advanced, non-invasive imaging technique that captures detailed blood flow dynamics in the retina and choroid with high resolution. Compared to traditional angiography, OCTA provides richer pathological information about the microvascular circulation in living tissues (Figure 1). It enables clear visualization of the retinal microvascular network—including superficial and deep vascular complexes as well as the choriocapillaris—offering clinicians critical insights into disease states and treatment responses (D’Agostino Sr et al., 2008; De Carlo et al., 2015; Germanese et al., 2024; Daho et al., 2024).

Coronary angiography is widely regarded as the “gold standard” for diagnosing CAD (Collet et al., 2017). This invasive procedure involves the insertion of a specialized catheter into the coronary arteries, followed by the injection of a contrast agent to visualize the arterial lumen. It enables detailed assessment of vascular conditions such as stenosis, blockage, and thrombosis (McClintic et al., 2010; Collet et al., 2017; Hoffmann et al., 2006). Coronary angiography provides critical information regarding the presence, location, severity, and extent of arterial narrowing, which is essential for determining appropriate treatment strategies—whether through interventional procedures, surgery, or medication (Roberts et al., 2008). Despite its diagnostic value, coronary angiography carries certain risks, including allergic reactions and vascular injury, which can pose significant concerns, especially for older adults (Hoffmann et al., 2005; Muhlestein et al., 2014). Additionally, it is a costly technique, making it more suitable for symptomatic patients requiring diagnostic confirmation, rather than for routine screening of asymptomatic individuals. In contrast, OCTA offers a noninvasive, cost-effective alternative. It has been demonstrated that the microvascular structures of the retina and heart share numerous similarities, and changes in retinal microvasculature are closely linked to cardiovascular health (Phan et al., 2015; Hubbard et al., 1999; Wong et al., 2002; 2008). OCTA enables the precise quantification of retinal vascular features such as vessel density, bifurcation count, fractal dimension, and the area of the foveal avascular zone (FAZ) (Wu et al., 2023; Nishida et al., 2023). These metrics serve as indicators of cardiovascular status and may help predict future disease risk. As such, OCTA imaging holds promise as a valuable and economical tool for the early screening of CAD.

In this study, we extracted quantitative parameters from OCTA images to assess the characteristics of retinal microvasculature and the FAZ. We then analyzed the relationship between these retinal microvascular changes and the risk of CAD. The goal of this research is to explore the potential of OCTA-based imaging as a noninvasive tool for early screening and risk assessment of CAD.

## 2 Related work

Modern multimodal ophthalmic imaging—paired with rapidly advancing artificial-intelligence (AI) algorithms has transformed the eye into a practical, non-invasive “window” on systemic vascular health. High-resolution fundus photography and optical coherence tomography angiography (OCTA) capture retinal microvascular features (e.g., vessel caliber, tortuosity, branching geometry) that mirror pathophysiological changes elsewhere in the body (Spaide et al., 2018; Panwar et al., 2016). Deep-learning models now exploit these rich image cues to detect subtle biomarkers of CAD and other cardiovascular disorders with accuracy that rivals, and sometimes exceeds, conventional clinical risk scores (Gupta and Reddy, 2021; Rim et al., 2021).

Richard et al. first hinted at an eye-kidney-vascular nexus by describing patients with albuminuria who also suffered vision loss, thereby linking systemic microvascular injury to ocular changes (Nishida et al., 2023). In today’s landscape—where cardiovascular disease (CVD) accounts for more than 30% of global deaths (Estimates, 2016)—early identification of at-risk individuals is critical. The American College of Cardiology’s ASCVD Risk Estimator Plus incorporates demographics, blood pressure, and lipid levels, yet its external calibration and discrimination remain sub-optimal (Arnett et al., 2019). This shortfall has sparked interest in retinal biomarkers that can be obtained quickly, painlessly, and at scale.

Poplin et al. demonstrated that a convolutional-neural-network model applied to fundus photographs could infer age, sex, smoking status, systolic and diastolic blood pressure, glycated hemoglobin, and even predict major adverse cardiac events (Poplin et al., 2018). An embedded soft-attention mechanism highlighted vascular arcades and the optic disc as key regions, lending biological plausibility to the predictions. Complementing this work, Hanssen et al. combined static vessel-diameter measurements with dynamic dilatory responses to derive a more holistic index of endothelial function and cardiovascular risk (Hanssen et al., 2022).

Alarmingly, CAD incidence is rising in younger populations. Ziegler et al. linked major atherosclerotic risk factors—hypercholesterolemia, hypertension, smoking, diabetes—to reduced capillary-density reserves, suggesting that microcirculatory rarefaction may precede clinical disease (Ziegler et al., 2019). Using OCTA, Zhong et al. showed that CAD patients have significantly lower retinal blood-flow density and thinner retinal layers, with metrics correlating to the severity of angiographic stenosis (Zhong et al., 2022). Subsequent studies reported thinner choroidal layers, enlarged FAZ, and reduced central vascular density in CAD cohorts, underscoring OCTA’s potential as a surrogate marker for disease burden and progression (Aschauer et al., 2021; Matulevičiūtė et al., 2022).

The eye–heart connection extends to congenital heart disease (CHD). Patients with CHD frequently present with retinal artery narrowing, atypical arteriovenous crossings, and other structural anomalies that impair vision. OCTA analyses by Li et al.

ed markedly reduced perfusion densities in both superficial and deep vascular plexuses among individuals with cyanotic CHD, compared with non-cyanotic counterparts and controls (Li et al., 2020). These findings reinforce the retina’s value for monitoring cardiovascular conditions across the lifespan.

Taken together, the existing studies have demonstrated that deep-learning analytics based retinal imaging becomes a powerful adjunct to CAD risk assessment. Fundus photographs enable rapid, low-cost population screening, while OCTA provides quantitative microvascular metrics that track disease severity. Ongoing efforts should prioritize multi-ethnic validation, longitudinal studies, and integration of ocular biomarkers with clinical and genomic data to build robust, personalized cardiovascular-risk models.

## 3 Methods

Our cohort included 747 participants, encompassing 966 eyes, including 415 controls and 332 patients diagnosed with CAD. Conducted at the Department of Cardiovascular Medicine, First Affiliated Hospital of Ningbo University, between October 2022 and October 2023, the study aimed to investigate the association between retinal microvascular alterations and CAD. All participants underwent coronary angiography and OCTA, and were stratified into CAD and control groups based on angiographic findings. Comprehensive clinical data were collected, including demographic variables (age, gender, height, weight), blood pressure parameters (systolic, diastolic, and pulse pressure), medical history (hypertension, hyperlipidemia, renal disease, central nervous system disorders, and diabetes), and lifestyle factors (smoking and alcohol consumption). Body Mass Index (BMI) was calculated from routine physical examination data. Inclusion criteria required participants to be over 18 years of age, possess complete clinical and imaging records, and have no history of congenital heart disease. Informed consent was obtained from all subjects prior to coronary angiography. The study protocol received ethical approval from the Ethics Committee of the Cixi Institute of Biomedical Engineering, Chinese Academy of Sciences, and the Ethics Committee of the First Affiliated Hospital of Ningbo University, and was conducted in strict accordance with the Declaration of Helsinki.

The OCTA images of all participants in the experiment are collected using AngioVue in the RTVue XR Avanti SD-OCT system (Optovue, USA). The device is scanned in a circular area with a diameter of 0.6 mm–2.5 mm centered on the central macular pits, resulting in a 3 × 3 ${\text{mm}}^{2}$ imaging area. The scanning speed is set at 70,000 times per second, producing binocular images of the superficial vascular complex (SVC) and deep vascular complex (DVC) with a resolution of 1,024 × 1,024 pixels for subsequent analysis. Images with signal intensity below seven are excluded from our data analysis.

For each OCTA image, we employed a pre-trained adaptive feature fusion multi-task network (VAFF-Net) to simultaneously segment retinal vessel and the FAZ (Hao et al., 2022). VAFF-Net comprises three principal components: a shared feature-extraction backbone, task-specific voting-gate module (VGM), and three dedicated prediction heads. The network ingests three en-face OCTA projections—the inner vascular complex (IVC), SVC, and DVC—and concurrently outputs retinal vessel segmentation, FAZ segmentation, and retinal vessel junction (RVJ) detection. A ResNet-50 backbone serves as the feature extractor; the three encoders share parameters except for their first convolutional layer, thereby preserving slab-specific characteristics (He et al., 2016). For each task, the VGM receives as input the concatenated outputs from the first layer of the three encoders. It subsequently produces a task-specific voting gate that adaptively selects informative features at two levels, across en-face images derived from different layer slabs, and across spatial locations within a single encoder. The proposed VAFF-Net employs a dynamic weighted average fusion (Liu et al., 2019) to integrate features for the three target tasks. Specifically, the retinal vessel segmentation and FAZ segmentation tasks are optimized using the standard binary cross-entropy (BCE) loss, while the RVJ detection task is trained using a combination of heatmap prediction loss and grid-based classification loss. VAFF-Net was evaluated on three OCTA datasets acquired with different devices (ROSE-O, ROSE-Z, and ROSE-H). Across all datasets, it consistently outperformed state-of-the-art single-task baselines and existing multi-task models, yielding superior Dice coefficient and balanced accuracy. These results underscore the proposed model’s robustness and enhanced generalisability under heterogeneous imaging conditions.

**FIGURE 2:**
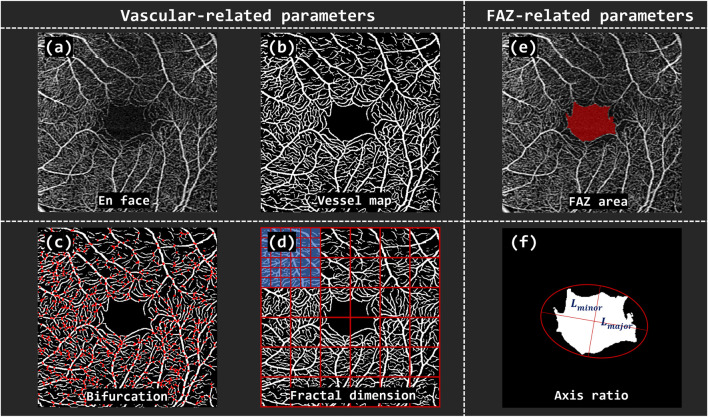
The overview of vascular and FAZ-related parameters. **(A)** *En face* OCTA image of the retina. **(B)** Binarized vessel map. **(C)** Vascular Bifurcation Number (VBN) extracted from the vascular network. **(D)** Vascular Fractal Dimension (VFD) calculated by grid-based box-counting method. **(E)** The defined FAZ area (FA). **(F)** FAZ Axis Diameter Ratio (FR) computed based on the major and minor diameters of the fitted ellipse.

To quantitatively characterize the retinal vessel and FAZ, a total of seven morphological parameters were extracted from OCTA images, including five vessel-related metrics and two FAZ-related metrics (Figure 2). The vessel-related parameters comprised Vascular Area Density (VAD), Vascular Length Density (VLD), Vascular Fractal Dimension (VFD), Vascular Tortuosity (VT), and Vascular Bifurcation Number (VBN). The FAZ-related parameters included FAZ Area (FA) and FAZ Axis Diameter Ratio (FR). Specifically, VAD was defined as the proportion of the retinal area occupied by perfused vasculature per unit area of the scan field $\left(mm^{2}\right)$ , reflecting the overall perfusion status. VLD represented the total length of retinal vessel centerlines normalized by the scan area, providing a measure of vascular coverage. VFD quantified the complexity and self-similarity of the vascular branching pattern using fractal geometry; lower VFD values indicate rarefaction or loss of capillary networks. VT assessed the curvature of retinal vessels, where increased tortuosity may suggest microvascular wall abnormalities, impaired hemodynamics, or tissue hypoxia. VBN denoted the number of bifurcation points within the vascular network, determined by analyzing the connectivity of centerline pixels. For the FAZ, FA referred to the total area of the avascular zone, while FR was defined as the ratio of the major to minor axes of an ellipse fitted to the FAZ, capturing its geometric regularity and potential distortion. Together, these parameters provide a comprehensive profile of retinal microvascular morphology and are valuable for assessing systemic vascular health.

To identify potential biomarkers associated with CAD, we conducted independent t-tests to compare OCTA-derived parameters between the control and CAD groups. These analyses were performed separately for data from both eyes combined, as well as for the left and right eyes individually. Additionally, we examined participants’ lifestyle factors and medical histories to explore potential risk factors for CAD. A p-value of less than 0.01 was considered statistically significant for all analyses, indicating a robust threshold for detecting meaningful differences.

## 4 Results

Table 1 summarizes the demographic, clinical, and lifestyle differences between the control group and the CAD group. Overall, individuals in the CAD group were older and predominantly male compared to controls. Health-related variables revealed significantly higher rates of hypertension and diabetes among CAD patients, reinforcing the established roles of these conditions as major risk factors for CAD. In addition, lifestyle factors such as smoking and alcohol consumption differed markedly between groups, with CAD patients showing higher prevalence of both behaviors, further suggesting their strong association with CAD onset.

**TABLE 1:** Results of demographic data.

Variable	Control (N = 415)	CAD (N = 332)			
	Mean (SD)	Mean (SD)	OR	95% CI	P
Age	57.95 (10.2)	61.35 (10.73)	1.032	1.017 to 1.047	<0.001
No of females (%)	51.08	34.64	0.507	0.377 to 0.683	<0.001
Hypertension (%)	46.02	66.57	2.335	1.732 to 3.148	<0.001
Diabetes (%)	13.49	28.92	2.608	1.804 to 3.769	<0.001
Smoking (%)	28.26	42.60	1.884	1.388 to 2.556	<0.001
Drinking (%)	18.84	24.17	1.373	0.965 to 1.953	<0.05

Table 2 displays comparisons of OCTA-derived parameters between the CAD and control groups. In terms of vascular metrics, CAD patients exhibited reduced VAD, VLD, and VBN compared to controls. For FAZ-related parameters, the FA showed a decreasing trend in the CAD group; however, substantial variability limited its statistical significance. Changes in other measured parameters did not reach statistical significance.

**TABLE 2:** Comparison of OCTA-derived parameters between healthy and patient eyes (n indicates the number of eyes analyzed).

Variable	Control (n = 566)		CAD (n = 400)	
	Mean	SD	Mean	SD
VAD	27.99	3.00	27.04	3.43
VLD	9.88	1.08	9.50	1.27
VFD	1.73	0.02	1.72	0.03
VT	1.85	0.17	1.83	0.16
VBN	210.33	40.22	217.39	41.78
FA	9796.07	2882.32	9447.27	3113.23
FR	1.12	0.06	1.13	0.80

**FIGURE 3:**
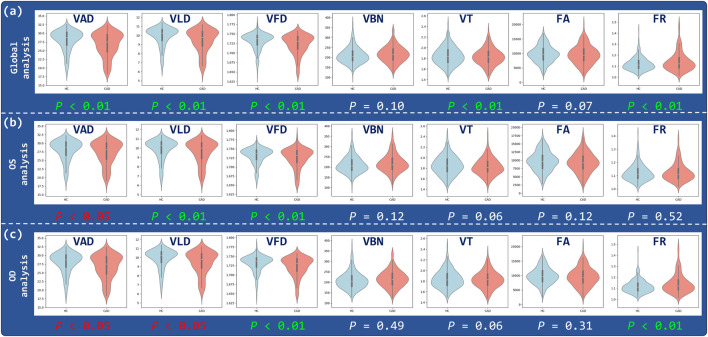
Correlation analysis of OCTA parameters. Violin plots illustrate the distributions of VAD, VLD, VFD, VBN, VT, FA, and FR for three analysis regions: **(a)** Global, **(b)** OS, and **(c)** OD.

The results of the OCTA parametric analysis are shown in Figure 3, illustrates the distribution differences of various retinal vascular parameters between the CAD group and the control group. In the combined binocular (Global analysis), VAD, VLD, VFD, VT and FR showed statistically significant differences between the two groups (P<0.01) . Further eye-specific analysis reveals that the left eye (OS) demonstrates greater sensitivity than the right eye (OD). Notably, in the OS analysis, VAD, VLD, and VFD all reach statistical significance, while VAD is not significant in the global analysis but becomes significant in the left eye, suggesting enhanced sensitivity of OS to microvascular changes. Although VLD and VFD remain significant in the OD analysis, the overall parameter sensitivity is lower compared to OS. These findings suggest that the left eye may serve as a more responsive indicator in the early screening of CAD and should be prioritized in subsequent modeling and diagnostic strategies.

**FIGURE 4:**
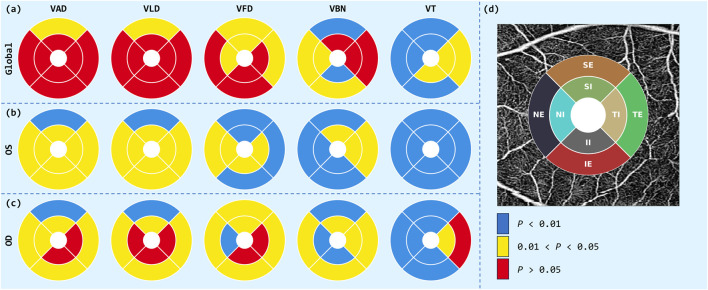
ETDRS grid analysis of OCTA parameters. Circular diagrams illustrate the distributions of VAD, VLD, VFD, VBN and VT for three analysis regions: **(a)** Global, **(b)** OS, and **(c)** OD. **(d)** ETDRS template: eight-sector grid superimposed on an en-face OCTA image.

The statistical analysis of the ETDRS grid is shown in Figure 4, where there was significant spatial heterogeneity in the differences in retinal vascular parameters between the case and control groups. From the Global analysis, parameters such as VAD, VLD, and VFD showed extensive red areas, indicating significant differences in most areas (P<0.05) . This suggests that the overall statistical power was enhanced when data were collected from both eyes, showing robust group level differences. For VBN and VT, most regions showed moderate statistical significance (0.01<P<0.05) and no significance (P>0.05) .

The analysis results of lifestyle factors and medical history are presented in Table 3. Individuals with a history of hypertension, kidney disease, central nervous system disorders, or cerebrovascular disease were found to have a significantly higher risk of CAD (P<0.01) , with hypertension showing the strongest association. Additionally, lifestyle habits such as smoking and alcohol consumption were also positively associated with an increased risk of CAD.

**TABLE 3:** Comparison of lifestyle habits and disease history.

	HLP	KD	NSD	CVD	AF	HF
p-value	0.517	<0.001	<0.01	<0.01	0.4049	0.2307

	SBP	DBP	PR (pm)	Weight	Height	BMI
p-value	0.6323	0.0443	0.0167	0.0149	0.0176	0.4697

## 5 Discussion

In this study, OCTA was used to analyze retinal vascular morphology and vessel density in CAD patients and controls. Microvessel density was reduced and vessel morphology was impaired in the patient group compared to the control group, suggesting microvascular damage in the pathogenesis of CAD.

Possible explanations for this phenomenon are as follows. On the one hand, coronary atherosclerotic heart disease may be a type of heart disease caused by disturbance of coronary blood flow, the underlying cause of which is arterial stiffness and impaired vascular endothelial function. Coronary artery is the artery responsible for supplying blood to the heart, and its lesions may cause systemic vascular dysfunction. With the progression of arteriosclerosis, the structure and function of microvessels are gradually damaged. As a tissue with a very high demand for oxygen, the hypoxic environment of the retina can activate a variety of pathophysiological reactions, leading to the damage and degeneration of retinal vessel. Under hypoxia, the microvessels of the retina are prone to deformation, occlusion, and damage, which reduce their vascular density. In addition, patients with CAD are often accompanied by oxidative stress. Excessive generation of oxygen free radicals can damage vascular endothelial cells and cause microvascular dysfunction. Under the influence of oxidative stress, retinal microvessels are prone to degenerative changes, including damage of vascular endothelial cells, thinning of capillaries, and reduction of blood flow, resulting in a decrease in microvessel density.

In our cohort the inter-group differences were consistently larger for OCTA parameters measured in the OS than in the OD. This asymmetry can be rationalised from a haemodynamic and anatomic standpoint. The ophthalmic artery supplying the OS arises from the left internal carotid, which itself branches directly from the left common carotid emerging from the aortic arch just distal to the left-coronary ostium. Consequently, the retinal microvasculature of the OS is exposed to essentially the same pulsatile pressure waves, shear-stress profiles and atherogenic milieu that promote plaque formation in the left coronary artery (LCA). When flow-limiting lesions develop in the LCA, comparable disturbances propagate along the ipsilateral carotid-ophthalmic axis; endothelial dysfunction and capillary rarefaction may therefore appear earlier or progress more rapidly in the OS, yielding larger OCTA-derived perfusion deficits relative to controls. Additional factors—such as lateralised autonomic innervation, ocular dominance, and subtle anatomical differences in ophthalmic-artery branching—may further accentuate the vulnerability of the OS. Together, these considerations provide a physiological basis for the more pronounced retinal alterations we observed in the left eye of patients with CAD.

## 6 Conclusion

In conclusion, this study explored the feasibility of OCTA in the early diagnosis of CAD by analyzing the changes of retinal vascular parameters in patients with CAD, and suggested that the differences between the left and right eyes should be considered when analyzing the changes of ocular vascular parameters. However, this study still has several limitations. First, the number of participants was still relatively small, and data were collected from only one eye in some participants. The study did not measure changes in retinal microvascular parameters over time or during disease progression. Second, axial length data, which is commonly used to identify myopia, were not obtained in this study, which may have affected the area captured by OCTA and thus introduced bias in the estimation of vascular parameters and subsequent analyses. Finally, more patient information needs to be collected to determine whether they are risk factors for CAD. These limitations can be optimized in future work.

## Data Availability

The raw data supporting the conclusions of this article will be made available by the authors, without undue reservation.
